# Towards Dissecting the Mechanism of Protein Phosphatase‐1 Inhibition by Its *C*‐Terminal Phosphorylation

**DOI:** 10.1002/cbic.202000669

**Published:** 2020-11-17

**Authors:** Francesca Salvi, Bernhard Hoermann, Javier del Pino García, Miriam Fontanillo, Rita Derua, Monique Beullens, Mathieu Bollen, Orsolya Barabas, Maja Köhn

**Affiliations:** ^1^ Genome Biology Unit European Molecular Biology Laboratory Meyerhofstraße 1 69117 Heidelberg Germany; ^2^ Signalling Research Centres BIOSS and CIBSS University of Freiburg Schänzlestraße 18 79104 Freiburg Germany; ^3^ Faculty of Biology University of Freiburg Schänzlestraße 18 79104 Freiburg Germany; ^4^ Laboratory of Biosignaling and Therapeutics Department of Cellular and Molecular Medicine KU Leuven Herestraat 49 3000 Leuven Belgium; ^5^ Laboratory of Protein Phosphorylation and Proteomics Department of Cellular and Molecular Medicine KU Leuven Herestraat 49 3000 Leuven Belgium; ^6^ SyBioMa KU Leuven Herestraat 49 3000 Leuven Belgium; ^7^ Structural and Computational Biology Unit European Molecular Biology Laboratory Meyerhofstraße 1 69117 Heidelberg Germany

**Keywords:** biomimetic synthesis, peptides, protein phosphatase-1 (PP1) regulation, protein semisynthesis, phosphorylation

## Abstract

Phosphoprotein phosphatase‐1 (PP1) is a key player in the regulation of phospho‐serine (pSer) and phospho‐threonine (pThr) dephosphorylation and is involved in a large fraction of cellular signaling pathways. Aberrant activity of PP1 has been linked to many diseases, including cancer and heart failure. Besides a well‐established activity control by regulatory proteins, an inhibitory function for phosphorylation (p) of a Thr residue in the *C*‐terminal intrinsically disordered tail of PP1 has been demonstrated. The associated phenotype of cell‐cycle arrest was repeatedly proposed to be due to autoinhibition of PP1 through either conformational changes or substrate competition. Here, we use PP1 variants created by mutations and protein semisynthesis to differentiate between these hypotheses. Our data support the hypothesis that pThr exerts its inhibitory function by mediating protein complex formation rather than by a direct mechanism of structural changes or substrate competition.

## Graphical abstract

**Interrogating a mechanistic black box**: Phosphoprotein phosphatase 1 (PP1) is among the major serine/threonine phosphatases and is involved in numerous pathways. Autoinhibition by phosphorylation of Thr320 at the *C*‐terminus leads to cell‐cycle arrest and neuronal defects. However, the mechanism underlying this regulatory phosphorylation site is unknown. Herein, we investigate potential mechanisms.

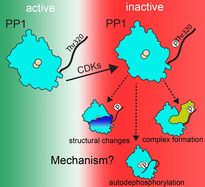

Phosphoprotein phosphatase‐1 (PP1) is a key player in cell signaling, catalyzing more than one third of phospho‐serine (pS)/‐threonine (pT) dephosphorylation reactions in eukaryotes.^[1]^ A tight regulation is therefore essential, and *in vivo* the enzymatic activity of PP1 is restrained and specified by the formation of holoenzymes.[^2^, ^3^] Besides this layer for control of enzymatic activity, acute activity control of PP1 is also provided by post‐translational modifications (PTMs), especially by phosphorylation of a conserved threonine residue (numbering 320 in PP1α, 316 in PP1β and 311 in PP1γ, Figure 1a) in the proline‐rich PxTPP sequence of the disordered *C*‐terminal tail (C‐tail).[^4^, ^5^, ^6^, ^7^, ^8^, ^9^] The phosphorylation of this Thr by cyclin‐dependent kinases (CDKs) represents an important control mechanism for cell‐cycle progression and neuronal differentiation, and it has been associated *in vivo* with an inhibitory effect on PP1.[^5^, ^6^, ^7^, ^8^, ^10^, ^11^, ^12^] Indeed, the timely phosphorylation of the PxTPP sequence by CDK2 during the G1 to S phase transition correlates with an accumulation of phosphorylated retinoblastoma protein, which is an important substrate of PP1.^[5]^ This phosphorylation step is crucial to enter and maintain S phase, as demonstrated by the fact that the phosphorylation‐resistant alanine mutant of PP1 causes a G1 arrest.^[13]^ Another relevant time‐point for phosphorylation of the PxTPP sequence during cell cycle is M phase, where different mitotic substrates of PP1 were described, such as histone H3 and proteins implicated in chromosome segregation and cytokinesis.^[1]^ The importance of the timing of PP1 inhibition and reactivation for controlling mitotic progression was also demonstrated.^[14]^ It was hypothesized that exit from mitosis relies on the reactivation of PP1 by auto‐dephosphorylation, however detailed mechanistic studies on auto‐dephosphorylation were lacking.^[13]^ In the context of neuronal differentiation, stimulation of neurons through *N*‐methyl‐d‐aspartate (NMDA)‐receptor signaling was associated with neuron growth^[15]^ and regulated PxTPP sequence phosphorylation of PP1 by CDK5.^[16]^ However, the mechanism through which phosphorylation of the PxTPP sequence of PP1 reduces PP1 function is unclear. Several mechanisms have been proposed. First, phosphorylation might inhibit PP1 due to direct competition with other phosphorylated substrates for binding to the active site.[^8^, ^16^, ^17^] Second, phosphorylation of the PxTPP sequence could induce interactions of the intrinsically disordered C‐tail with PP1 on an allosteric site to induce inhibitory conformational changes. Third, phosphorylation of the PxTPP sequence could affect interactions of PP1 with other proteins. To address these possibilities, here we applied structural, semisynthetic, proteomic and biochemical methods to study the effect of phosphorylation of recombinant PP1α at Thr320 in the PxTPP sequence in a controlled setup.

**Figure 1:**
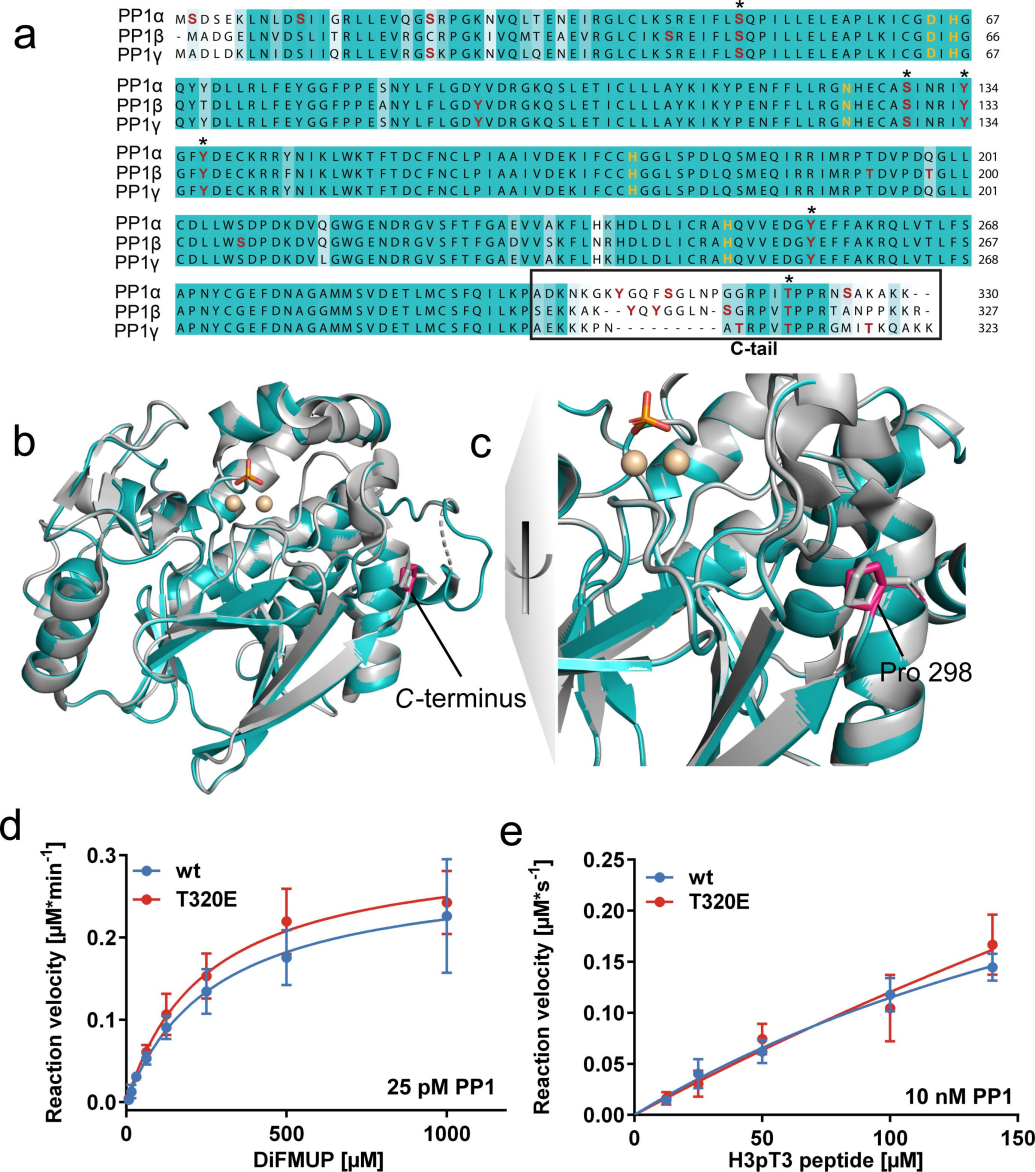
Structure and activity of PP1α‐T320E. a) Sequence alignment of the three human PP1 isoforms. The sequences were obtained from uniprot.org and aligned by using ClustalOmega. Color coding refers to sequence identity calculated in the software Jalview (v2.11.1.0). Known phosphorylation sites were extracted from phosphositeplus.org and are highlighted by red letters. Residues coordinating the metal ions in the active site are highlighted by orange letters. Asterisks highlight phosphorylation sites conserved across all three isoforms. b) Structural alignment of PP1α‐T320E (cyan) with a previously published structure of active Fe‐PP1α (6G0I)^[20]^ (gray). Metal ions in the catalytic center are depicted as spheres. P298, as the last visible amino acid in the structure, is highlighted in pink for PP1α‐T320E. c) Rotation and zoom of the aligned structures near P298. No electron density can be detected in either the wild‐type or phosphomimetic T320E mutants for residues following P298. d) 25 pM recombinant PP1α wt/T320E were incubated with 8–1000 μM DiFMUP substrate, and the activity was monitored by detecting the fluorescence of the product 6,8‐difluoro‐4‐methylumbelliferone (DiFMU). The assay was conducted in three independent biological repeats carried out in triplicate. Error bars represent SD of the mean. e) 10 nM recombinant PP1α wt/T320E was incubated with 12.5–140 μM H3pT3 peptide. The rate of dephosphorylation was measured by monitoring the secondary enzymatic conversion of purine nucleoside phosphorylase (PNP) with methylthioguanosine (MESG) and released phosphate to 2‐amino‐6‐mercapto‐7‐methylpurine by absorption at 360 nm. The assay was carried out twice in duplicate. Error bars represent SD of the mean. Activity was assessed by comparing slopes (wt 2.6^−6^±1.6^−7^ AU s^−1^ μM^−1^; T320E 3.0^−6^±2.6^−7^ AU s^−1^ μM^−1^).

In order to examine the role of PP1’s C‐tail we first inspected existing crystal structures. In the PP1 structures deposited in the Protein Data Bank (www.PDB.org)^[18]^ no electron density was observed for the residues in direct vicinity of T320, and in most cases the C‐tail even had to be removed to allow protein crystallization. An exception represents a single complex, in which residues 317–322 (RPITPPR) of PP1α become visible due to binding of the PP1 interactor ASPP2.^[19]^ This is consistent with the intrinsically disordered properties of the unphosphorylated tail in wild‐type PP1, and suggests a role for it in protein‐protein interactions. For the phosphorylated C‐tail, it is possible that the negative charges of pThr320 promote the formation of new interactions with another region of PP1α, thus inducing a closed, inactive protein conformation. To test this hypothesis, the T320E variant of the most abundant isoform PP1α was expressed, purified (Figure S1 in the Supporting Information) and crystallized following a previously reported protocol.^[20]^ The structure was solved at 1.9 Å to determine whether the insertion of a negative charge in the tail induces a conformational change or leads to its stabilization by binding to the catalytic protein core (Figure 1b,c, Table S1). Similar to previous structures, no electron density was observed for amino acids following P298 in PP1α containing Glu in position 320. The superposition of the main chain with the wild‐type crystal structure solved in our previous study^[20]^ highlighted no significant movement of the main chain (Figure 1b: root‐mean‐square deviation 0.110 on superposition of Cα atoms with PDB ID: 6G0I) and no significant differences in the position of side chains. These findings lead to the conclusion that mimicking of the negative charge at the phosphorylation site Thr320 does not alter the 3D structure of the catalytic core of PP1α and does not trigger structural reorganization of the disordered C‐tail.

Since no structural rearrangements could be observed for the PP1α T320E variant, we next investigated the effect of the additional negative charge on PP1α on its enzymatic activity. PP1α T320E was tested in parallel with wild‐type (wt) PP1α *in vitro*, using two different substrates and enzymatic assays. The first assay monitored PP1α activity on 6,8‐difluoro‐4‐methylumbelliferone phosphate (DiFMUP), whereas the second detected inorganic phosphate from dephosphorylation of a peptide carrying the sequence of the well‐established substrate site Thr3 of Histone 3 (H3pT3).[^21^, ^22^] We did not observe a significant change in the kinetic parameters between wild‐type and T320E enzyme in either of the two assays (Figure 1d,e). This shows that the introduction of a negative charge to mimic the phosphorylation of Thr320 does not measurably affect the enzymatic activity *in vitro*. This finding presents a contrast to the suggested mechanism for direct autoinhibition of PP1 by phosphorylation.[^7^, ^8^]

Site‐directed mutagenesis of Thr or Ser phosphorylation sites with negatively charged Asp or Glu is a widely used strategy to mimic phosphorylated residues.^[23]^ However, the negative charge of the side chain of Asp and Glu cannot fully account for all steric and electrostatic properties of pThr/pSer. Because of the reported *in vitro* auto‐dephosphorylation occurring in the required conditions for CDK2/cyclin A phosphorylation of recombinant PP1,^[24]^ hydrolyzable pThr obtained from *in vitro* phosphorylation of the recombinant protein is not suitable to study the effect of Thr320 phosphorylation on PP1 activity. Therefore, we designed a semisynthetic protein^[25]^ to create a non‐hydrolyzable version of PP1α‐pT320. Currently the best mimetic for pThr is the non‐hydrolyzable amino acid phosphono‐difluoromethylenealanine (*Pfa*) (Figure 2a).^[26]^ To insert *Pfa* at position 320, we expressed truncated PP1α‐Δ316 as intein fusion^[20]^ and coupled the *Pfa*‐containing synthetic C‐tail by native chemical ligation.[^25^, ^27^] Because protein semisynthesis creates a G316C mutation that is present in the final semisynthetic protein (Figure S2a), we tested whether the G316C mutation has an effect on PP1α activity. As shown in Figure S2b, incorporation of C in position 316 did not affect the functionality of PP1α. The C*‐*tail including *Pfa*320 was then synthesized by solid‐phase peptide synthesis (SPPS), purified, and characterized (see the supporting information) before coupling to PP1α‐Δ316 by native chemical ligation, which yielded the semisynthetic PP1α variants LigT320 and LigPfa320 (Figures 2a and S2c,d). We then tested the enzymatic activity of the semisynthetic *Pfa*‐PP1α (LigPfa320) in parallel with the unphosphorylated semisynthetic enzyme (LigT320) *in vitro*, using DiFMUP and H3pT3 as substrates as described above. Introducing *Pfa*320 did not result in reduced activity compared to the unphosphorylated semisynthetic PP1α, consistent with our observations using PP1α‐T320E (Figure 2b,c).

**Figure 2:**
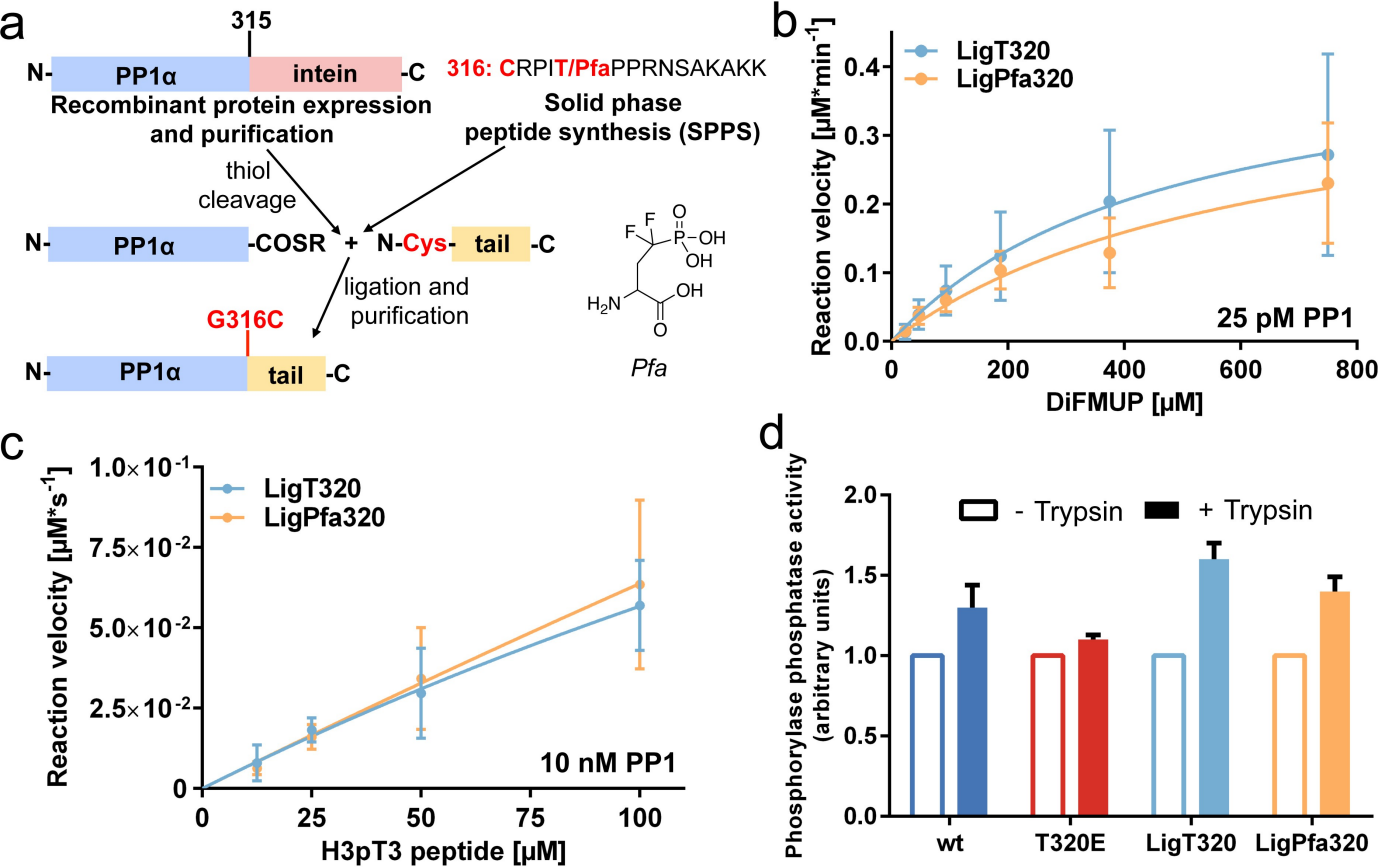
Generation and activity of semisynthetic PP1α‐*Pfa*320. a) Semisynthesis of PP1α‐*Pfa*320 (LigPfa320). To test the feasibility of this approach, a PP1α‐G316 C mutant (Figure S2a) was first tested for activity (Figure S2b). A shortened version of PP1, PP1α(7‐315) was then expressed and purified using an intein tag, followed by thiol cleavage to create the active thioester (see the Supporting Information). In parallel, the tail sequence containing Thr320 or *Pfa*320 was synthesized by SPPS, purified, and characterized (see the Supporting Information). Recombinant PP1α(7‐315) and the respective peptide were then linked by native chemical ligation. Besides a size shift (Figure S2c), intact mass analysis confirmed the integrity of the semisynthetic proteins (Figure S2d). b) Recombinant protein (25 pM) was incubated with 23–750 μM DiFMUP substrate, and the enzymatic activity was monitored as in Figure 1d. The assay was conducted in three independent biological repeats in duplicate. Error bars represent SD of the mean. c) 10 nM semisynthetic protein was incubated with 12.5–100 μM H3pT3 peptide. The rate of dephosphorylation was measured as in Figure 1e. The assay was carried out twice in triplicate. Error bars represent SD of the mean. Both proteins show an approximately twofold lower activity than the recombinant proteins in Figure 1e due to the ligation buffer. d) The effect of trypsin on the activity of recombinant PP1α (wt and T320E mutant) and semisynthetic PP1α variants (LigT320 and LigPfa320) towards glycogen phosphorylase *a* was tested. After preincubation of 4 nM PP1 with 54 ng μL^−1^ trypsin (5 min, 30 °C), the substrate phosphorylase *a* was added at 10 μM, which is close to the *K*_m_.^[29]^ The experiment was carried out three times in triplicate. Results are shown as mean±SEM.

To further confirm the lack of catalytic impairment in *Pfa*‐PP1α (LigPfa320), we also tested the enzymatic activity after a limited trypsinolysis. As demonstrated previously, trypsin hydrolyzes the C terminus of PP1 but cannot readily digest its catalytic core.[^28^, ^29^] When PP1α phosphomimetic variants T320E and LigPfa320 were tested against untreated protein, we generally detected a slight increase in phosphatase activity toward the substrate phosphorylase *a* by prior trypsinolysis, consistent with a previously observed inhibitory effect of the C‐tail on the phosphatase activity. However, the effect of trypsinolysis was similar for the wt, T320E, LigT320 and LigPfa320 variants, indicating that the phosphomimetic proteins were inhibited to a similar extent by their C‐tail as the wt and LigT320 enzymes. (Figures 2d and S3). PP1 can also be inactivated by slowly induced conformational changes, as seen for example upon incubation with inhibitor‐2, which renders the catalytic core of PP1 sensitive to proteolysis.^[30]^ However, the introduction of phosphate‐mimicking PP1 mutations did not appear to induce such an inactivating conformational change, as the phosphatase activity of these PP1 variants was not destroyed by trypsinolysis. This conclusion is consistent with the crystal structure of PP1α‐T320E obtained (Figure 1b,c), which did not reveal structural rearrangements.

All experiments presented up to this point used a concentration ratio of enzyme versus substrate in the range of 1 : 10^3^–10^6^. In such a setup, due to the high dilution, PP1 is much more likely to dephosphorylate substrate molecules than to interact with other phosphatase molecules in *trans*, thus rather reporting on intramolecular autoinhibition. Since our data suggested that phosphorylation of Thr320 does not lead to inhibition of its own PP1 molecule in *cis*, we hypothesized that spatial constraints and tail length prohibit intramolecular inhibition of PP1 by pThr320 and that intermolecular interactions could instead lead to the proposed autoinhibition through pThr320 *in vivo*.[^8^, ^12^, ^15^] To test this hypothesis and increase the likelihood of intermolecular interactions, assay setups with a high excess of pThr/*Pfa*‐320‐containing protein/peptide versus enzyme were examined.

In an *in vitro* activity assay quantifying release of inorganic phosphate upon peptide dephosphorylation, PP1α was able to use a peptide carrying the sequence of its own phosphotail as substrate but with very low catalytic efficiency (Figure 3a). Twenty times more PP1α enzyme had to be used with the C‐tail peptide compared to the histone 3 tail (H3pT3)‐derived peptide to obtain detectable phosphate release on the same 96‐well plate with uniform detection settings. Despite this increased phosphatase amount, the C‐tail peptide GRPIpTPPRNSAKAKK was still dephosphorylated with 7.5‐fold lower efficiency compared to the H3pT3 peptide. These results indicated that the *C*‐terminal tail is a poor substrate for intermolecular auto‐dephosphorylation, and were substantiated on the protein level using the PP1αD64N‐pT320 mutant (Figure S4). Substituting Asp64 in the catalytic center with Asn has previously been shown to yield a PP1 variant with strongly decreased activity,^[31]^ thereby preventing auto‐dephosphorylation and dephosphorylation of glycogen phosphorylase *a*. Titration of increasing amounts of PP1 (mixture of isoforms, purified from rabbit skeletal muscle) into this reaction led to efficient dephosphorylation of glycogen phosphorylase *a*, without any evidence for intermolecular dephosphorylation of pThr320 on PP1αD64N‐pT320, providing further evidence that PP1α‐pThr320 is a rather poor substrate of the PP1 catalytic subunit alone for auto‐dephosphorylation in *trans*, irrespective of the PP1 isoform. To investigate whether dephosphorylation‐independent autoinhibition of PP1α via pThr320 could take place in *trans*, we tested the dephosphorylation of the PP1 substrate glycogen phosphorylase *a* in the presence of T320/*Pfa*320‐containing C‐tail peptides (Figure 3b). Titration of peptides did not significantly affect the dephosphorylation of phosphorylase *a* by PP1α. Thus, the C‐tail peptides did not appear to compete with the substrate for PP1 recognition or to inhibit PP1α independently of an active site interaction. This result was supported by a competition experiment on the protein level when using equimolar amounts of unphosphorylated PP1α alone (single‐PP1 assay) compared to unphosphorylated PP1α or LigT320 mixed with T320E or Lig*Pfa*320 mutants, respectively, to dephosphorylate glycogen phosphorylase *a* (mixed‐PP1 assay, Figure 3c). Again, no difference in dephosphorylation activity was observed. All the above results clearly indicated that the PP1 autoinhibition is not directly carried out by the catalytic subunit alone. We therefore sought to investigate whether the interaction between the PP1C‐tail and an additional protein in a pThr320‐dependent manner could explain the autoinhibition of PP1. To this end, *Pfa*‐containing C‐tail or the unphosphorylated control peptide were incubated with cell lysate, followed by MS‐based identification of binding proteins (Table S2). However, these experiments did neither reveal interactors above levels of background binding proteins, nor proteins binding the PP1C‐tail in a phosphorylation‐dependent manner with high fold changes.

**Figure 3:**
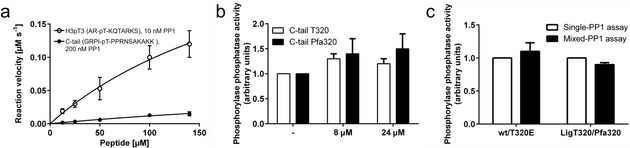
Testing the C‐tail as a substrate for inhibitory auto‐dephosphorylation. a) 10 or 200 nM recombinant PP1α (1‐330) was incubated with 12.5–140 μM H3pT3 peptide (AR‐pT‐KQTARKS) or C‐tail peptide (GRPI‐pT‐PPRNSAKAKK), respectively. The rate of dephosphorylation was measured by monitoring the secondary product 2‐amino‐6‐mercapto‐7‐methylpurine by absorption at 360 nm. The assay was carried out twice with three technical repeats. Error bars represent SD of the mean. b) The glycogen phosphorylase *a* (10 μM) activity of recombinant wt PP1α was measured after the preincubation of 4 nM PP1α with the indicated peptide concentrations for 10 min at 30 °C. Results are shown as the mean±SEM of three independent assays each measured in duplicate. c) The glycogen phosphorylase *a* (10 μM) phosphatase activity of 4 nM PP1α wt/T320E or 4 nM PP1 α LigT320/LigPfa320 (single‐PP1 assay) as compared to the phosphatase activity of 2 nM PP1α wt+2 nM PP1‐T320E or 2 nM PP1α LigT320+2 nM PP1αLigPfa320, respectively (mixed‐PP1 assay). Results are shown as the mean±SEM of three independent assays, each measured in duplicate.

PP1 plays a role in numerous signaling pathways, regulated by interacting proteins and by phosphorylation of its own C‐tail at residue T320.[^5^, ^8^, ^13^, ^15^] The importance of regulating PP1α‐pThr320 has been demonstrated for cell‐cycle progression and neuronal stimulation of NMDA receptors.[^13^, ^14^, ^15^] However, despite the clear inhibitory function of PP1α‐pThr320, the underlying mechanism is unclear. The data presented herein clearly indicate that the mechanism of pThr320‐mediated PP1 autoinhibition goes beyond the previously assumed models of direct conformational changes or substrate competition without complex partners. Of note, while we observed very low auto‐dephosphorylation activity, it is possible that in cells holoenzymes could enhance this activity. According to our findings, the phosphorylation of Thr320 is also not likely to lead to a folding of the *C*‐terminal tail and its binding to the PP1 catalytic core. Consequently, our findings suggest a mechanism involving pThr320‐specific recruitment of (a) protein(s), which then inhibit PP1 directly or through an additional (covalent) modification, such as promoting oxidation of the Fe^2+^ in the catalytic center,^[20]^ or crosstalk between PTMs. Our C‐tail‐pulldown experiments also suggest that for a potential pThr320‐specific binding protein, besides interaction with the phosphorylated tail, additional binding to the core of PP1 or to a PP1 holoenzyme is needed for a stable interaction in order to mediate PP1 inhibition upon Thr320 phosphorylation. PP1 is known to be regulated by more than 200 proteins^[2]^ and modified by PTMs on multiple residues (Figure 1a), and it therefore seems likely that the autoinhibitory function of pThr320 is executed indirectly through PP1 holoenzyme formation or a complex interplay between PTMs. Interestingly, in the recent crystal structure of a PP1 complex with detectable electron densities for PP1α residues 317–322, binding of the tumor suppressor and apoptosis‐stimulating protein of p53 2 (ASPP2) depended on interactions with the PP1 catalytic core and on binding of the ASPP2‐SH3 domain to the PP1C‐tail.^[19]^ However, contrary to a pThr‐specific binding, the affinity of this interaction is likely changed upon phosphorylation of Thr320, because phosphomimetic mutations of the corresponding threonine residue in PP1 led to dissociation of ASPP proteins.^[32]^ This offers another complex scenario of indirect PP1 inhibition upon phosphorylation through dissociation of substrate‐targeting regulatory proteins, leading to PP1 not recognizing its substrates anymore.^[22]^

In conclusion, the structural, semisynthetic and biochemical approaches taken here have clearly shown that, contrary to previous assumptions, the inhibitory effect of the phosphorylation of the *C*‐terminal PxTPP sequence is due to an indirect mechanism, involving complex protein‐protein interactions. Therefore, future efforts should be focused on dissecting the possible cellular mechanisms of PP1 inhibition through C‐tail phosphorylation.

## Conflict of interest

The authors declare no conflict of interest.

## Supporting information

As a service to our authors and readers, this journal provides supporting information supplied by the authors. Such materials are peer reviewed and may be re‐organized for online delivery, but are not copy‐edited or typeset. Technical support issues arising from supporting information (other than missing files) should be addressed to the authors.

Supplementary
