# Supplementary material for: Towards Dissecting the Mechanism of Protein Phosphatase‐1 Inhibition by Its C‐Terminal Phosphorylation
Source: Chembiochem. 2020 Nov 17;22(5):834–8. doi: 10.1002/cbic.202000669 (PMC7984433; doi:10.1002/cbic.202000669)
Supplement: Supplementary file 1 — Supplementary [file CBIC-22-834-s001.pdf]

ChemBioChem

Supporting Information

Towards Dissecting the Mechanism of Protein Phosphatase-1 Inhibition by Its *C*-Terminal Phosphorylation

Francesca Salvi, Bernhard Hoermann, Javier del Pino García, Miriam Fontanillo, Rita Derua, Monique Beullens, Mathieu Bollen, Orsolya Barabas, and Maja Köhn*

Supporting Table 1………………………..………………………………….… p. 2
Supporting Figure 1…….………………….……....…………………….……... p. 3
Supporting Figure 2……………………….……...…………………..…….... p. 3
Supporting Figure 3……………………………………………………………... p. 4
Supporting Figure 4……………………………………………………………... p. 4
Analytical data of Fmoc-Pfa and the C-tail peptides.……………………………... p. 5–9
^{1}H-NMR Spectrum of Fmoc-Pfa………… ………………….…....………………… p. 5
^{19}F-NMR Spectrum of Fmoc-Pfa………… ……………….……....………………… p. 5
^{13}C-NMR Spectrum of Fmoc-Pfa………… ……………….……....………………… p. 6
^{31}P-NMR Spectrum of Fmoc-Pfa………… ……………….……....………………… p. 6
HPLC trace and ESI-MS spectrum of Fmoc-Pfa……...…………………….……….. p. 7
HPLC trace of the wt C-tail peptide……………………..…………..……….. p. 7
ESI-MS spectrum of the wt C-tail peptide……………………..……………….. p. 8
HPLC trace and ESI-MS spectrum of the Pfa C-tail peptide……………….……… p. 8
HPLC trace and ESI-MS spectrum of the purchased H3pT3 peptide…..……….. p. 9
HPLC trace and ESI-MS spectrum of the purchased C-tail phosphopeptide…. p. 10
Experimental procedures………………………………………………………. p. 11–17
Accession Codes…………………………………………………………………. p. 17
Supporting references…………………………………………………………. p. 18

Supporting Table 1. Summary of crystallographic data and refinement statistics.

**Data Collection**	
Space group	$P22_12_1$
a, b, c (Å)	38.55, 68.75, 127.85
α, β, γ (°)	90.00, 90.00, 90.00
Wavelength (Å)	0.9677
Resolution	36.22 – 1.90 (1.94 – 1.90)[a]
R_{merge} (%)	5.7 (74.4)
R_{meas} (%)	6.6 (86.6)
R_{pim} (%)	3.3 (43.3)
I/σ (*I*)	11.0 (1.50)
Completeness (%)	97.3 (98.9)
Redundancy	3.4 (3.5)
Number of observations	91468 (6056)
CC (1/2) (%)	99.9 (60.3)
**Refinement**	
Resolution (Å)	36.22 – 1.90
Number of reflections total / free set	26637 / 1286
R_{work}/R_{free} (%)	15.95 / 19.43
Rmsd bond lengths (Å) / angles (°)	0.011 / 1.181
Average B-value (Å^2)	43.0
Ramachandran favored (%)	96
Ramachandran outliers (%)	0

[a] Numbers in parentheses show the statistic for the highest resolution shell.

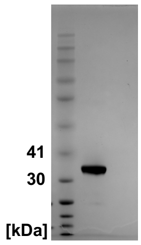

Supporting Figure 1. Coomassie stained gel of the purified PP1α T320E protein.

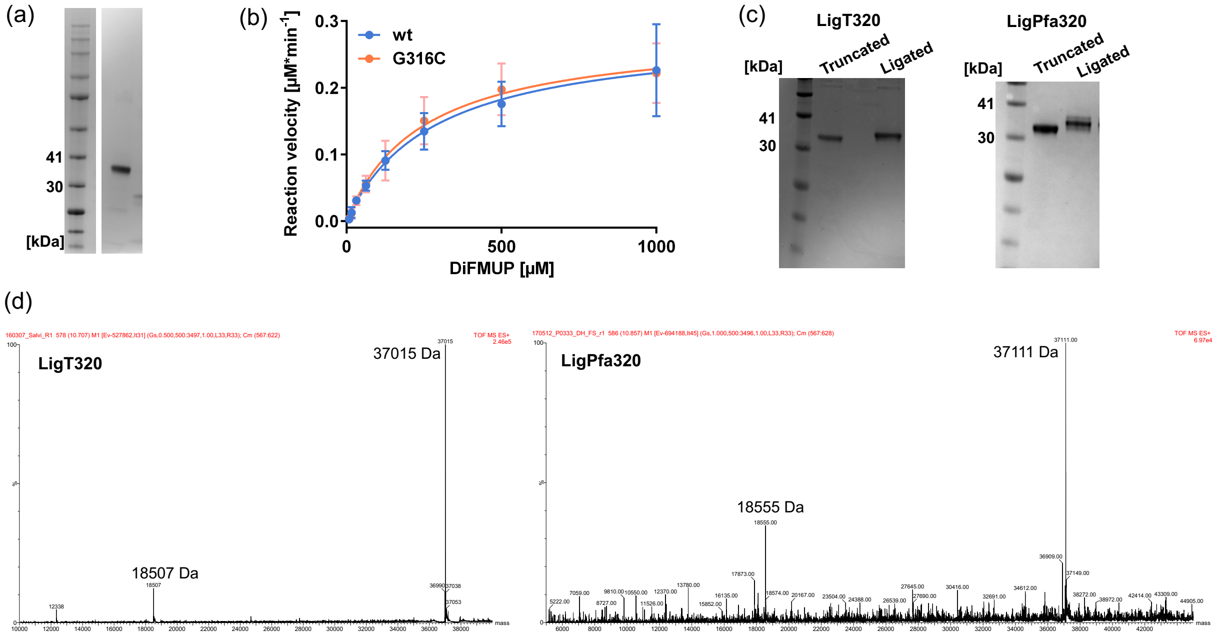

Supporting Figure 2. Control experiments and characterization of semisynthetic proteins. a) Coomassie stained gel of the purified PP1α G316C protein. b) 25 pM recombinant PP1α wt/G316C were incubated with 8–1000 µM DiFMUP substrate and the enzymatic activity was monitored by detecting the fluorescence of the product. The assay was carried out in three independent biological repeats each in triplicates. Data points and error bars represent mean ± SD. c) Coomassie stainings of semisynthetic PP1α. Recombinantly expressed and purified unligated PP1α (as *C*-terminal thioester, "truncated") compared to the semisynthetic proteins LigT320 and LigPfa320 ligated to the respective C-tail peptide ("ligated"). d) ESI-TOF-MS analysis of the semisynthetic proteins depicted as "ligated" in c).

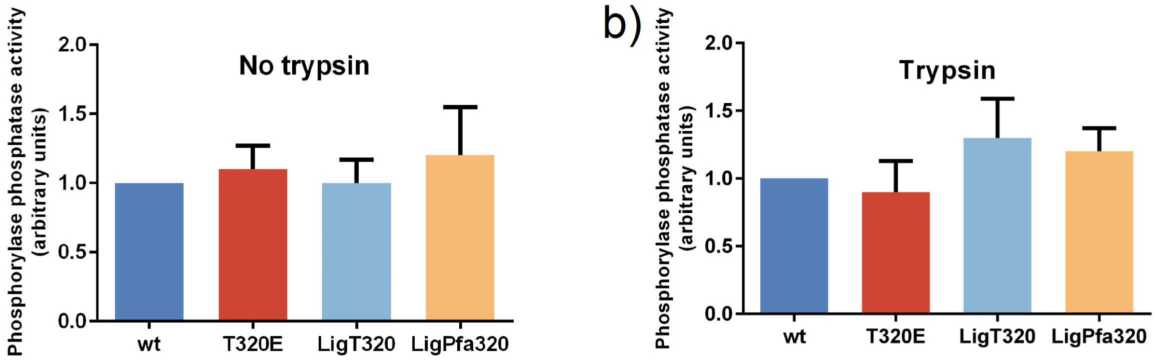

Supporting Figure 3. The effect of trypsin on the activity of recombinant PP1α (wt and T320E mutant) and semisynthetic PP1α variants (ligation with nonphosphorylated peptide LigT320, and phosphomimetic peptide Lig*Pfa*320) towards glycogen phosphorylase *a* was tested. The graphs are based on the same raw data as shown in Fig. 2d. Here, PP1 activity is normalized to wt in order to assess relative changes in phosphatase activity. After preincubation of 4 nM PP1 with 54 ng*μl^{-1} Trypsin (5 min, 30°C), the substrate glycogen phosphorylase *a* was added. Results are shown as means ± SEM (n = 3, technical triplicates each).

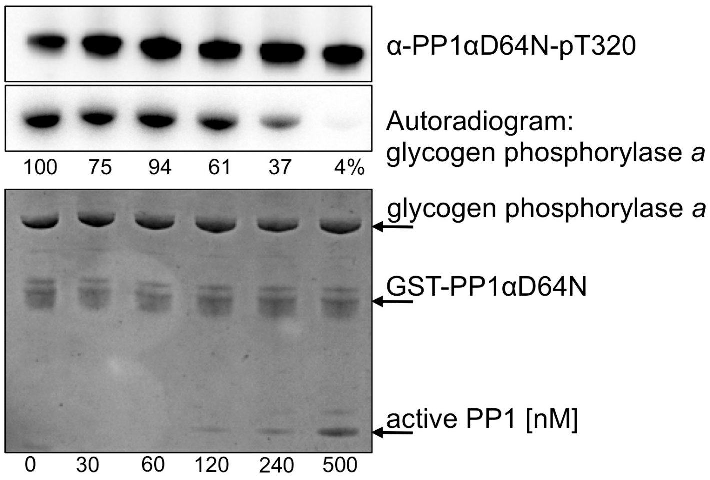

Supporting Figure 4. Titration of native (active) PP1 leads to dephosphorylation of glycogen phosphorylase *a*, but not of inactive PP1α-D64N-pT320. Enzymatically impaired recombinant GST-PP1α-D64N was phosphorylated by CDK2/Cyclin A on Thr320 and incubated with 0.5 μM glycogen phosphorylase *a*. Active PP1 purified from rabbit muscle was added at the indicated concentrations. Results were obtained by Western Blotting using a site-specific PP1α-pThr320 antibody, autoradiogram for phosphorylase *a* phosphorylation and Coomassie staining for monitoring amounts of proteins. Representative images of two repeats are shown.

Analytical Data of Fmoc-Pfa and the two C-tail peptides

1. NMR data

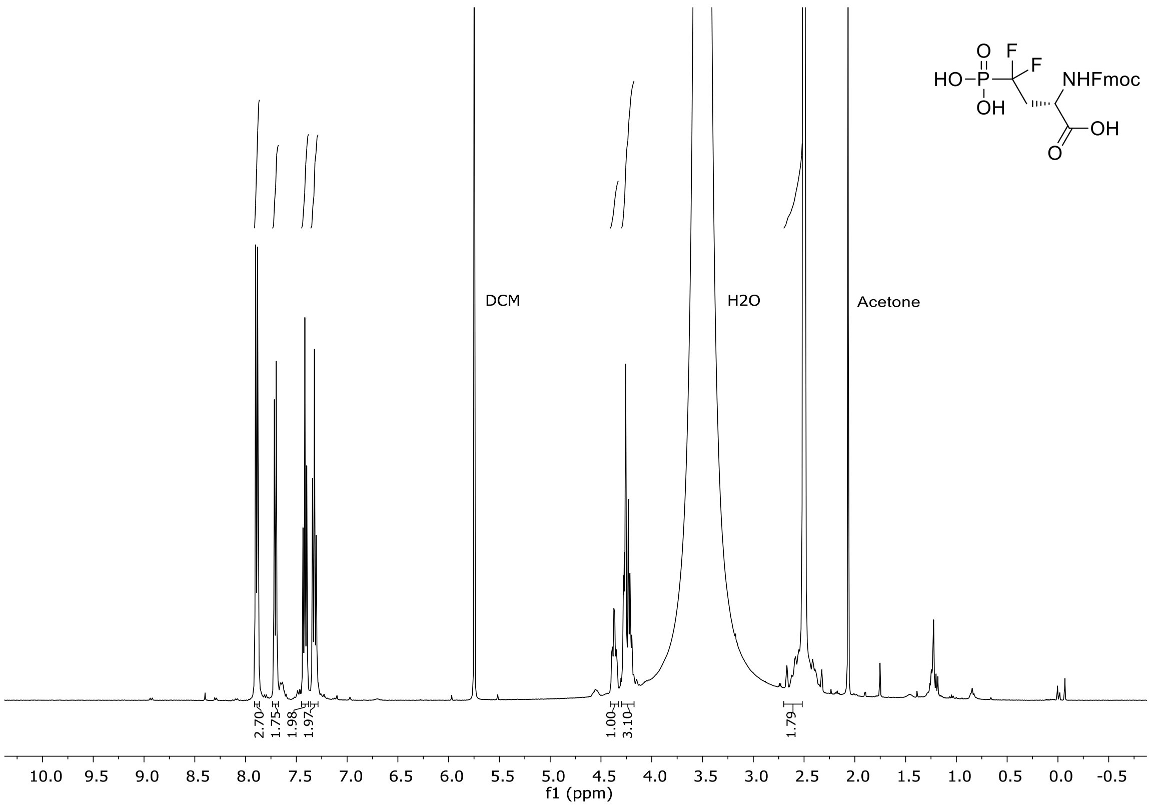

^1H spectrum of Fmoc-Pfa (not purified).

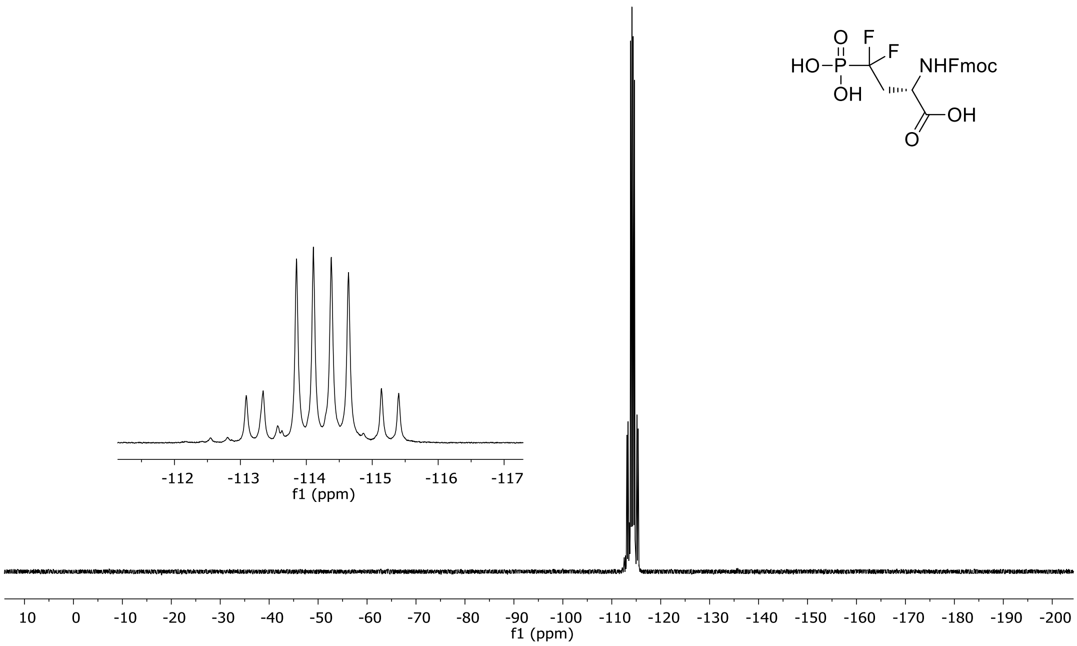

^{19}F spectrum of Fmoc-Pfa (not purified).

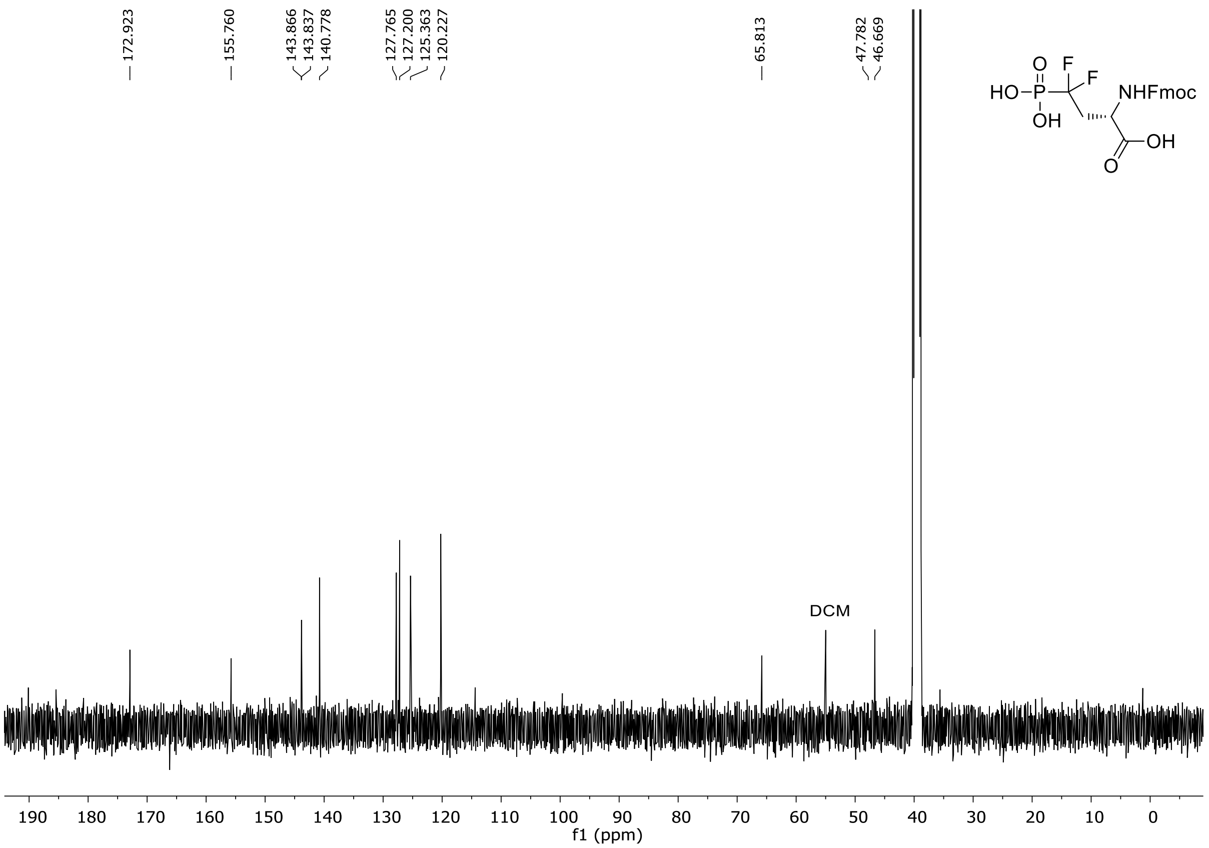

^{13}C spectrum of Fmoc-Pfa (not purified).

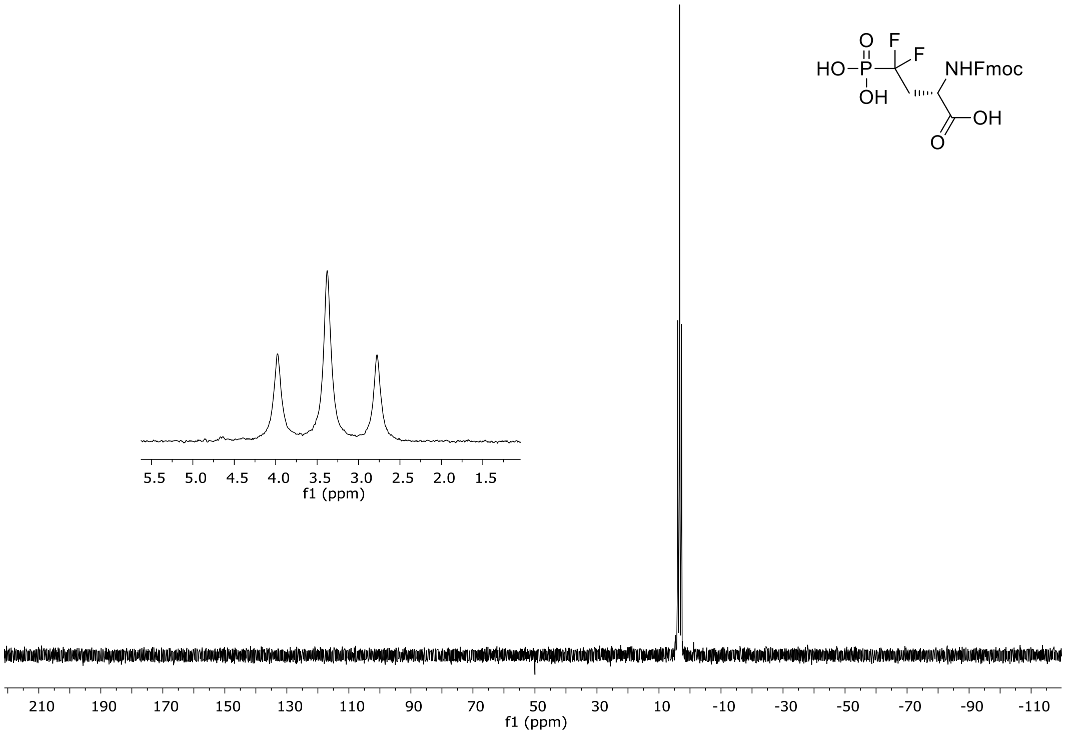

^{31}P spectrum of Fmoc-Pfa (not purified).

2. **HPLC-MS data**

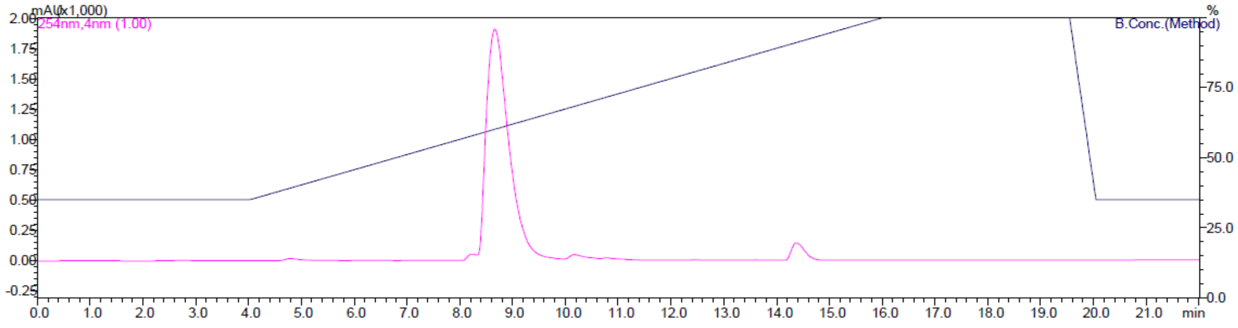

HPLC trace of Fmoc-Pfa (not purified).

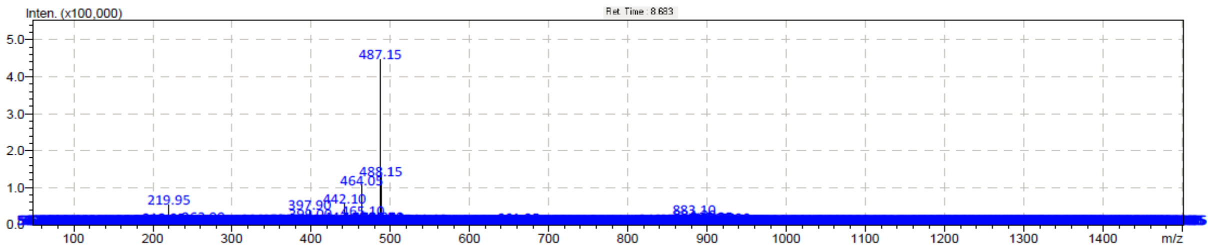

ESI-MS spectrum of Fmoc-Pfa (not purified). M + H^+ = 486.29 (calculated, disodium salt), 487.15 (found).

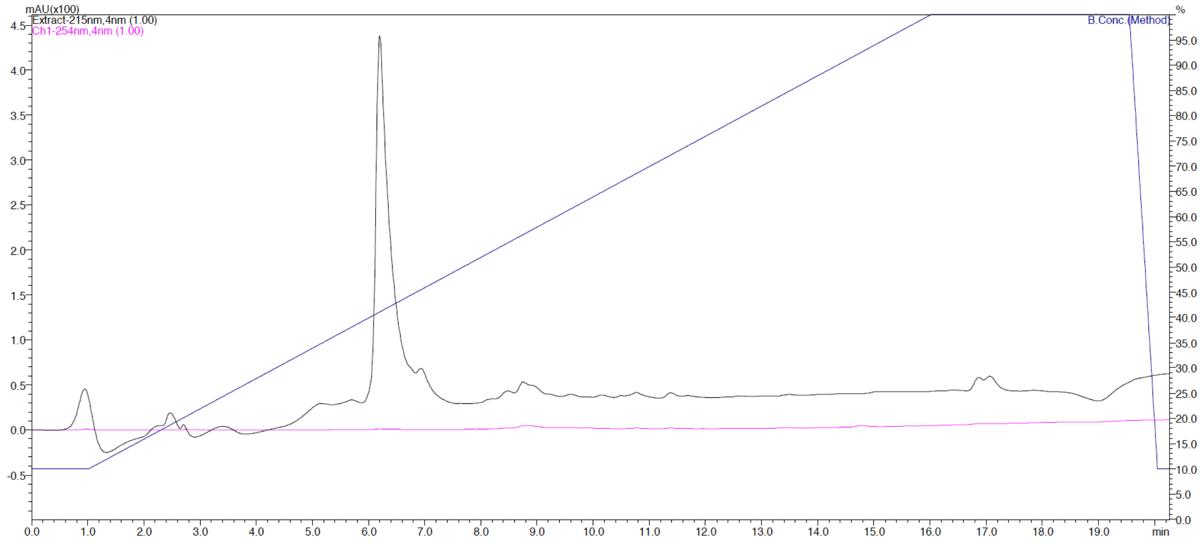

HPLC trace of the wt C-tail peptide.

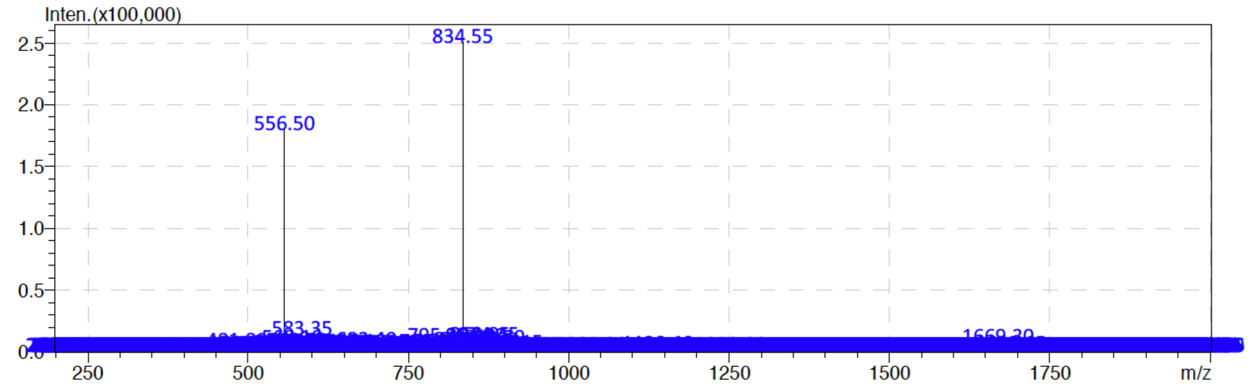

ESI-MS spectrum of the wt C-tail peptide. M + 2H$^+$ = 833.98 (calculated), 834.55 (found); M + 3H$^+$ = 556.32 (calculated), 556.50 (found).

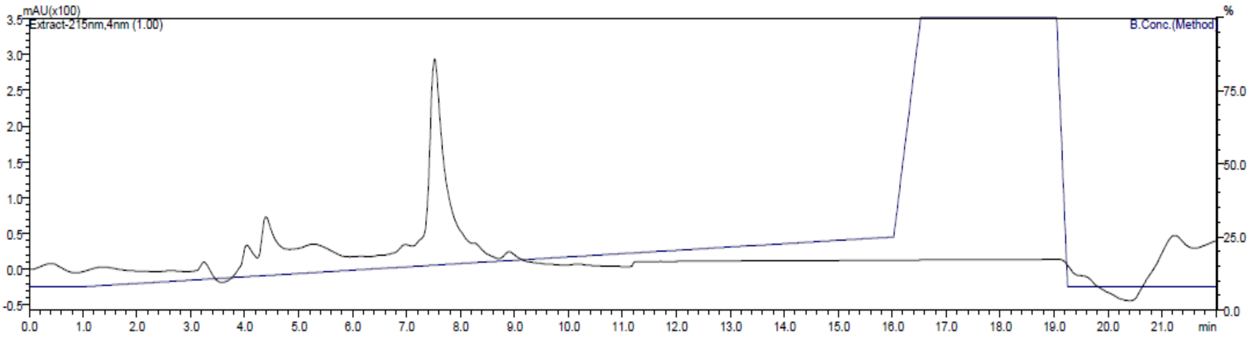

HPLC trace of the Pfa C-tail peptide.

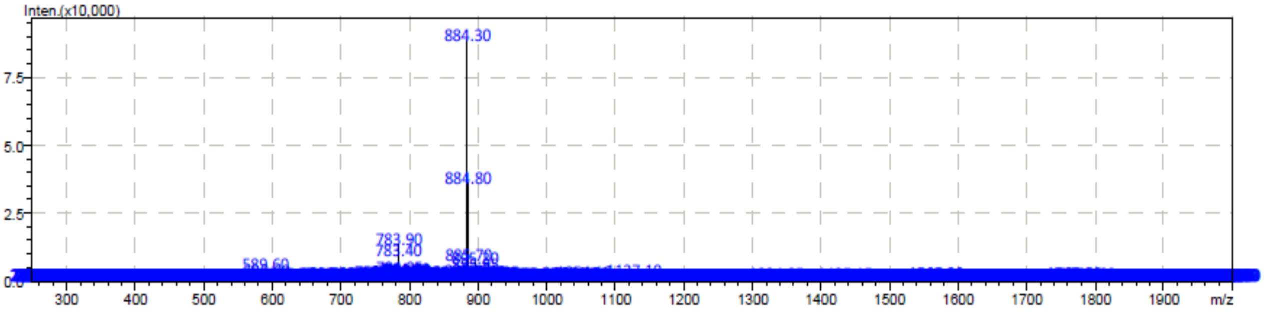

ESI-MS spectrum of the Pfa C-tail peptide. M + 2H$^+$ = 883.95 (calculated), 884.30 (found).

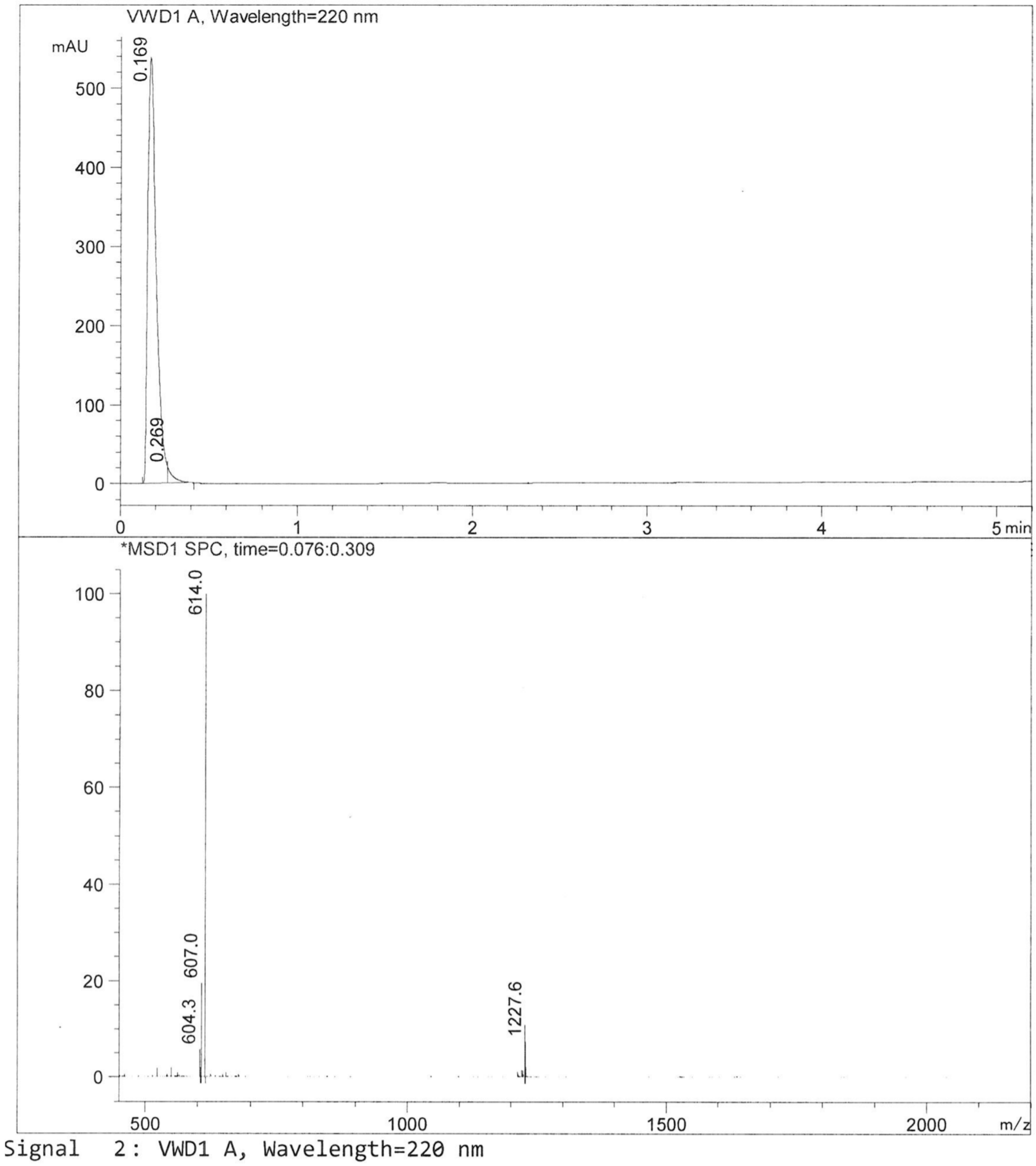

HPLC (top) and ESI-MS (bottom) data of the H3pT3 peptide purchased from JPT Peptide Solutions. M + H^+ = 1227.3 (calculated), 1227.6 (found); M + $2H^+$ = 614.2 (calculated), 614.0 (found).

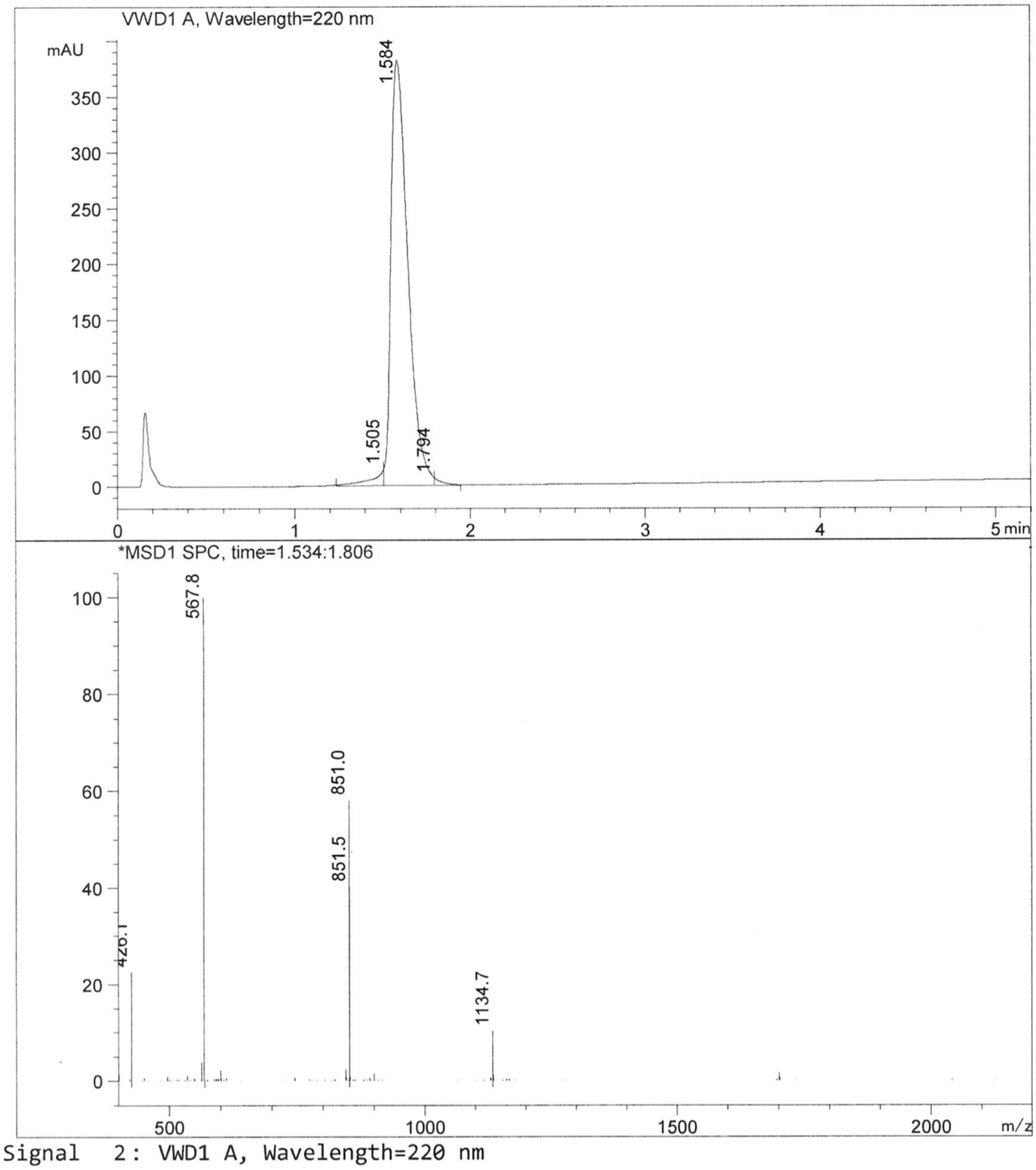

HPLC (top) and ESI-MS (bottom) data of the phosphorylated C-tail peptide purchased from JPT peptide solutions. $M + 2H^+$ = 851.5 (calculated), 851.0 (found); $M + 3H^+$ = 568.0 (calculated), 567.8 (found); $2M + 3H^+$ = 1134.9 (calculated), 1134.7 (found).

Experimental Section

Protein production and crystallization of T320E variant. Site-directed mutagenesis was carried out using the construct pTXB1-PP1α as template, which codes for full length PP1α(1-330) with one cleavable His-tag at the N terminus and one cleavable intein-tag at the *C*-terminus. For enzymatic assays, recombinant wild-type and T320E mutant PP1α were expressed in medium supplemented with $MnCl_2$ and purified as described previously.[1] For crystallization, a purification protocol with addition of iron instead of manganese was used, as described before for Fe-PP1α wild-type.[1] Prior to crystallization, the recombinant protein Fe-PP1α T320E was further purified by size-exclusion chromatography using a HiLoad 16/600 Superdex 200 column (GE Healthcare) equilibrated in 50 mM Tris-Cl pH 7.5 RT, 500 mM NaCl, 5 mM β-mercaptoethanol and concentrated to 6.9 mg*mL^{-1}. Crystals were grown at room temperature, using the sitting drop vapor diffusion technique with the precipitant solution containing 28% w/v PEG 3350, 0.1 M TRIS-Cl pH 8.0 RT, 1 M LiCl. Prior to the diffraction experiment, crystals were cryoprotected with the addition of 25% glycerol and flash frozen in liquid nitrogen. The dataset was collected at the ESRF beamline ID30A-3 and processed with XDS[2] and Aimless[3]. The phase problem was solved by molecular replacement in Phaser[4] (CCP4 suite[5]), using the previously published crystal structure of wild-type Fe-PP1α as model (PDB code 6G0J)[1]. The model was refined with Refmac5[6] and Phenix[7]. Manual building and adjustment was performed with Coot[8]. This dataset was deposited in the PDB database with the ID 6ZK6 and data collection and refinement statistics are presented in Supporting Table 1.

Chemical reagents and general methods. Solvents were purchased and used as received from the following suppliers: dichloromethane (DCM) from VWR; *N*,*N*-dimethylformamide (DMF), acetic acid anhydride (Ac_2O) from Merck; *N*,*N*-diisopropylethylamine (DIPEA) from abcr; *N*-methyl-2-pyrrolidone (NMP), diethylether, piperidine and pyridine from ROTH; acetonitrile (ACN) ≥ 99.9% HPLC grade, HPLC water LCMS chromasolv and methanol (MeOH) ≥ 99.9% HPLC grade from Sigma-Aldrich; anhydrous *N*,*N*-dimethylformamide (dry DMF) 99.8% from Alfa Aesar; dimethylsulfoxide-d6 (DMSO-d_6) 99.8% and chloroform-d ($CDCl_3$) from deutero GmbH. Reagents were used without further purification from the following commercial sources: Fmoc-Ala-OH, Fmoc-Cys(Trt)-OH, Fmoc-Pro-OH, Fmoc-Ile-OH, Fmoc-Arg(Pbf)-OH, Fmoc-Lys(Boc)-OH, Fmoc-Ser(OtBu)-OH, Fmoc-Asn(Trt)-OH

and H-Lys(Boc)-2-chlorotrityl resin from Merck Novabiochem; 2-(1H-benzotriazol-1-yl)-1,1,3,3-tetramethyluronium hexafluoro phosphate (HBTU) and *O*-(Benzotriazol-1-yl)-*N,N,N′,N′*-tetramethyluronium tetrafluoroborate (TBTU) from Bachem; triisopropylsilane (TIPS), from Alfa Aesar; hydroxybenzotriazole (HOBT) from MOLEKULA; trifluoroacetic acid (TFA), from ROTH; and 1-hydroxy-7-azabenzotriazole (HOAT) and Fmoc-homo-Arg(Pbf)-OH from GL Biochem (Shanghai) Lt; bromo-trimethyl-silane (TMS-Br) from Sigma-Aldrich. Fmoc-phosphonodiethyl-difluoromethylene alanine was purchased from Enamine.

^{1}H, ^{13}C ^{19}F and ^{31}P NMR spectra were recorded using Bruker UltraShield Avance III HD 400 MHz using Topspin 3.2 as acquisition software and MestReNova 10.0 to analyse the data. All chemical shifts are quoted in ppm and are reported using solvent as an internal standard: ^{1}H NMR: 2.50 ppm (DMSO-d6); ^{13}C NMR: 39.51 ppm (DMSO-d6). 2D COSY and HSQC and ROESY spectra were used to assist NMR signal assignments. Automatic synthesis of linear peptides was performed with a MultiSynTech Syro I Parallel Peptide Synthesis System using plastic reactors with 5 ml TF frit (from Multisyntech). Manual synthesis of peptides on solid phase and cleavage from the resin was performed using 5 mL or 2 mL reactors with TF frit and plastic plunges (from Multisyntech). Analytical HPLC runs (1.1 mL*min^{-1}) and purifications (5 mL*min^{-1}) were performed on a Shimadzu HPLC-MS 2010EV Evolution system with a reversed phase column from Macherey-Nagel (NUCLEODUR C18pyramid VP250/10 5μm particle size or NUCLEODUR 100-5C18ec 250/4.0) with a UV/Vis detector operating at λ = 215 and 254 nm. As mobile phases, ACN/0.05% TFA and H_2O/0.05% TFA were used. A Waters MALDI micro MX-TOF mass spectrometer was used using a saturated solution of alpha-cyano-4-hydroxycinnamic acid in ACN/H_2O with 1% TFA as matrix.

Synthesis of Fmoc-Phosphono-difluoromethylenealanine (Fmoc-Pfa). 104 mg (0.21 mmol) Fmoc-phosphonodiethyl-difluoromethylene alanine were dissolved in 1 mL of dry DCM and cooled in an ice bath. Under argon, TMS-Br (500 μl, 3.79 mmol, 18 equiv) was added dropwise. The solution was stirred and allowed to warm to room temperature overnight. Solvents were removed *in vacuo* and the process was repeated once more. After that, a mixture of H_2O/ACN (10%) was added and the solution was stirred for 2.5 h at room temperature. Then, solvents were removed *in vacuo* and the procedure was repeated once more. The white solid (43% overall yield considering weight) was used without further purification for the synthesis of the

peptide on solid phase. HPLC analysis t_R = 8.68 min (C18pyramid, 35–100% ACN/H_2O 0.05% TFA in 16 min); ^{1}H NMR (400 MHz, DMSO-d6): δ 7.89 (d, *J* = 7.5 Hz, 2H, aryl-H Fmoc), 7.71 (d, *J* = 7.5 Hz, 2H, aryl-H Fmoc), 7.42 (dd, *J* = 7.4 Hz, 2H, 2 aryl-H Fmoc), 7.32 (dd, *J* = 7.4 Hz, 2H, 2 aryl-H Fmoc), 4.37 (td, *J* = 8.7, 3.5 Hz, 1H, CαH), 4.24 (m, 3H, CH-CH_2 Fmoc), 2.49 (m, CH_2-CF_2) ppm; ^{13}C NMR (101 MHz, DMSO-d6): δ 172.9 (COOH), 155.8 (CONH), 143.9 (2 aryl-C Fmoc), 143.8 (2 aryl-C Fmoc), 140.8 (CF_2), 127.8 (2 aryl-C Fmoc), 127.2 (2 aryl-C Fmoc), 125.4 (2 aryl-C Fmoc), 120.2 (2 aryl-C Fmoc), 65.8 (CH_2 Fmoc), 47.8 (CαH), 46.7 (CH Fmoc) ppm (CF_2 not obviously visible); ^{19}F NMR (376 MHz, DMSO-d6): δ -114.24 (dddd, *J* = 96.055, 98.596, 98.596, 98.596 Hz, CF_2) ppm; ^{31}P NMR (162 MHz, DMSO-d6): δ 3.38 (t, *J* = 97.3 Hz, $PO(OH)_2$) ppm; MS (ESI-pos): m/z calcd. for $C_{19}H_{17}F_2NNa_2O_7P^+$: 486.29 $[M+H]^+$; found: 487.15.

PP1 tail synthesis. The two peptide tails CRPI(<u>T</u>)PPRNSAKAKK (wt) and CRPI(<u>*Pfa*</u>)PPRNSAKAKK (Pfa) for the production of semisynthetic PP1α were synthesized on a Lys(Boc) preloaded 2-chlorotrityl resin (0.78 mmol*g^{-1}) using a MultiSynTech peptide synthesizer. The wt-peptide was fully synthesized on the synthesizer whereas the *Pfa*-peptide was synthesized on the synthesizer only until Pfa incorporation. In general, Fmoc-amino acids (5 equivalents) were coupled by double couplings in the presence of DIPEA (10 equivalents), HOBt (0.2 M), and HBTU (5 equivalents) in DMF using the standard protocol. The coupling time was 40 minutes each and was followed by capping with 10% Ac_2O in pyridine. Fmoc deprotection was carried out using 40% piperidine in DMF for 3 minutes and then again 20% piperidine in DMF for 14 minutes. After cleavage from the resin using 95% TFA / 2.5% TIPS / 2.5% water, the peptides were precipitated in cold diethyl ether, and purified by reverse phase chromatography with a C18 pyramid column from Macherey-Nagel (see above).

To incorporate Fmoc-*Pfa* the coupling of this building block and of the following amino acids Ile, Pro, Arg, and Cys was carried out manually to the peptide PPRNSAKAKK connected to the resin. A test cleavage by shaking in the cleavage cocktail (TFA/TIPS/H_2O 95:2.5:2.5) for 3 hours and measurement of the product formation in the HPLC-MS was carried out before attaching Fmoc-*Pfa*. Then, Fmoc-*Pfa* (37.7 mg, 85.47 µmol, 2.5 equivalents) was manually attached to 45 mg of peptide connected to the resin using TBTU (5 equivalents), HOAT (5 equivalents) and DIPEA (10 equivalents) in dry DMF under argon and shaking overnight. After

washings with DMF and DCM, again a test cleavage was carried out to confirm the successful coupling. After washing steps with DMF and DCM, Fmoc deprotection was carried out as detailed above. Next, the four final amino acids were attached on the peptide synthesizer using 5 equivalents of Fmoc-Ile (double coupling for 2 hours), Fmoc-Pro (double coupling for 1 hour), Fmoc-Arg (double coupling of 2 hours) and Fmoc-Cys (double coupling for 1 hour), HBTU (5 equiv), HOBT (0.2 M) and DIPEA (10 equiv) in DMF. Finally, Fmoc deprotection by piperidine was followed by capping using 10% Ac_2O in pyridine. The cleavage from the resin and the peptide purification was carried out as described above. HPLC analysis for the *Pfa*-peptide: t_R = 7.5 min (C18pyramid, 8-25% ACN/H_2O 0.05% TFA in 15 min); MS (ESI-pos): m/z calcd. for $C_{71}H_{127}F_2N_{25}O_{21}PS^+$: 883.96 $[(M+2H)/2]^{2+}$; found: 884.30. HPLC analysis for the wt-peptide: t_R = 6.2 min (C18pyramid, 10-100% ACN/H_2O 0.05% TFA in 15 min); MS (ESI-pos): m/z calcd. for $C_{71}H_{127}N_{25}O_{19}S^+$: 833.98 $[(M+2H)/2]^{2+}$, 556.32 $[(M+3H)/]^{3+}$; found: 834.55, 556.50.

Protein production of semisynthetic (T/*Pfa*)-PP1α. The ligation procedure, illustrated in Figure 2a, introduces the presence of a cysteine residue at position 316 *in lieu* of the glycine residue of the wild-type sequence. In order to evaluate the choice of the ligation site the variant G316C was created by site-directed mutagenesis to investigate how well tolerated is the introduction of a cysteine at this position on the activity of PP1α. The construct pTXB1-PP1α(7–315) was used to overexpress a truncated version of PP1α fused with intein at the *C*-terminus[1]. After lysis by Emulsiflex of 12.5 g of bacterial cell pellet in lysis buffer (50 mM HEPES-Na pH 7.0, 1 M NaCl, 1 mM $MnCl_2$, 0.2% v/v tween 20, 0.1 mM PMSF, 0.5 mM $MgCl_2$, Roche protease inhibitor, 125 U benzonase) the cleared supernatant was loaded on a chitin column equilibrated in high salt buffer (50 mM HEPES-Na pH 7.0, 1 M NaCl, 1 mM $MnCl_2$, 0.2% v/v tween 20). One step of stringent wash with the high salt buffer was performed followed by an extensive wash in lower salt buffer (50 mM HEPES-Na pH 7.0, 300 mM NaCl, 0.2% v/v tween 20). The chitin column with the PP1α(7-315)-intein precursor bound was then equilibrated with cleavage buffer (50 mM HEPES-Na pH 7.0, 300 mM NaCl, 0.2% v/v tween 20, 100 mM MESNA), sealed and incubated overnight at 4℃. After incubation, the cleaved PP1α(7-315) thioester was eluted from the chitin column and $MnCl_2$ was added to a final concentration of 1 mM. PP1α(7-315) thioester was concentrated to 2.6 mg*mL^{-1} and ligated to the *Pfa*-tail peptide in 1:3 ratio in presence of 100mM MESNA and 0.5 mM TCEP. After

overnight incubation more peptide and fresh reducing agents were added to the ligation reaction, which was further incubated at 4 °C and proceeded to completion in 4 days at 4℃. The ligated protein was purified by ionic exchange using a Mono Q column equilibrated in 40 mM TRIS-Cl, 100 mM NaCl, 5% glycerol, 1 mM DTT and 1 mM $MnCl_2$ with a gradient to 40% of high salt buffer with 1 M NaCl. Purity based on SDS-PAGE (Supplemental) is at least 95% and the yield was 0.9 mg of purified *Pfa*-PP1 from 12.5 g of bacterial pellet (corresponding to 2 L of bacterial growth). Intact mass measurement (Supporting Fig. 2d) confirmed the successful ligation and did not detect residual truncated PP1α(7–315) thioester. The truncated PP1 was also ligated with the threonine-tail peptide in order to create the unphosphorylated reference threonine-PP1 by following the same procedure and the identity was confirmed by intact mass measurement (Supporting Fig. 2d).

Enzyme activity assays. *In vitro* activity assays were performed in 96 well plates with a Tecan Infinite M1000 PRO. All assays were conducted at 25℃ in a final volume of 100 µL with three independent experiments. Each experiment was designed in duplicate or triplicate, and the compared enzymes (T320E or G316C variants versus full length wild type and semisynthetic Thr-PP1 versus *Pfa*-PP1) were analyzed in parallel in the same plate. Activity assays with DiFMUP were conducted by monitoring the fluorescence of the product 6,8-difluoro-4-methylumbelliferone (DiFMU). The activity buffer was 50 mM imidazole, 10 mM NaCl, 2 mM DTT, 1 mM $MnCl_2$, 0.05% tween at a final pH of 7.4. The substrate DiFMUP was diluted in activity buffer so that the final concentration of DMSO was kept below 2%. The enzymatic activity was linear in respect to time and enzyme concentration. The final concentration of enzyme used was 25 pM and the substrate ranged from 8 to 1000 µM.

The activity assays with the H3pT3 peptide (sequence: AR-pT-KQTARKS) as substrate were performed with the EnzCheck Phosphate Assay (Thermo Fisher). In this assay the production of phosphate by PP1 is coupled to the reaction of purine nucleoside phosphorylase (PNP, 0.15 unit) with 200 µM methylthioguanosine (MESG) as substrate. The final product 2-amino-6-mercapto-7-methylpurine is detected by monitoring the absorbance at 360 nm. The enzymatic activity was linear with respect to time and enzyme concentration, and it was assessed that the amount of coupled enzyme was not limiting. Addition of the peptide substrate did not alter the pH of the activity buffer. The activity buffer was 20 mM TRIS-Cl, pH 7.5 RT, 100 mM

NaCl,1 mM DTT, 1 mM $MnCl_2$. The final concentration of enzyme used was 10 nM and the substrate ranged from 12.5 to 140 µM. The activity assay with the peptide carrying the sequence of the phosphorylated tail with sequence GRPI-pT-PPRNSAKAKK was performed with the EnzCheck Phosphate Assay (ThermoFisher) as described above for the assay with the H3pT3 peptide, but 200 nM PP1 had to be used to yield signal comparable to 10 nM PP1 incubated with the H3pT3 peptide.

Glycogen phosphorylase *a* assay and trypsin treatment. The phosphosubstrate glycogen phosphorylase *a* was prepared as described previously.[9] Bacterially expressed GST/His-tagged human PP1α (wild type and T320E mutant) and semisynthetic PP1α variant (see above) were diluted to 4 nM in dilution buffer (25 mM Tris-HCl at pH 7.5 incl. 0.1 mg*mL^{-1} BSA). The phosphatase activity was determined after incubation for 10 min at 30 °C in assay buffer (dilution buffer + 0.1 mM $MnCl_2$ + 2 mM DTT) from the rate of dephosphorylation of glycogen phosphorylase *a*, present at 10 µM representing the substrate concentration at K_m[10]. The extent of dephosphorylation was assessed from the released acid-soluble radioactivity (P_i). The phosphorylase phosphatase activity was assayed either as such or after preincubation with trypsin (54 ng*μL^{-1}) for 5 min at 30 °C. The action of trypsin was arrested by the addition of soybean trypsin inhibitor (104 ng*μL^{-1}).[10]

Phosphorylase *a* assay with PP1αD64N-pT320. Recombinant GST-PP1αD64N was purified as described before[11] and incubated for 1h at 30°C with Cdk2/cycl A in 25 mM Tris/HCl at pH 7.4, supplemented with 0.1 mg*ml^{-1} BSA, 1 mM DTT, 100 µM ATP and 2 mM $MgCl_2$. The phosphorylation reaction was stopped with 100 µM roscovitine. 2 µM of phosphorylated GST-PP1αD64N-pT320 was incubated for 20 min at 30°C with 0.5 µM glycogen phosphorylase *a* and various concentrations of native PP1 (0–500 nM), purified from rabbit skeletal muscle[12]. The reaction was stopped by boiling in sample buffer and the mixture was loaded on SDS-PAGE. The dephosphorylation of phosphorylase *a* was analyzed by autoradiography and the phosphorylation level of GST-PP1αD64N-pT320 was analyzed by immunoblotting with a phosphoPP1αT320-specific antibody (Cell Signalling #2581). Total protein levels were visualized by Coomassie staining.

C-tail peptide pulldown and mass spectrometry analysis

200 µg of the PP1 C-terminal peptides were coupled to 250 µg of maleimide-activated BSA (Thermo Fisher catalog number 77115) and BSA-coupled peptides

were coupled to 0.1 g of CNBr-activated sepharose (Sigma GE17-0430-01), according to the protocol of the manufacturers. A control-matrix was made by coupling 250 µg of free BSA to 0.1g of CNBr-activated sepharose.

BSA-peptide (or BSA alone as a control) affinity columns were incubated with HeLa cell mitotic lysates for 2h at 4°C and, after washing with lysis buffer (2x) followed by 200 mM ammonium bicarbonate (2x), were subjected to overnight on-bead trypsin digestion (1 µg of trypsin at 37 °C in a total of 150 µl containing 100 mM ammonium bicarbonate, 2.5 % acetonitrile and 0.01% ProteaseMax). The resulting peptides were desalted with C18 ZipTip pipette tips (Millipore) and subjected to high-resolution LC-MS/MS using an Ultimate 3000 Nano Ultra High-Pressure Chromatography (UPLC) system interfaced with a Q Exactive Hybrid Quadrupole-Orbitrap mass spectrometer via an EASY-spray (C-18, 50 cm) column (Thermo Fisher Scientific). Peptides were identified by MASCOT (Matrix Science) using Swiss-Prot/TrEMBL Homo sapiens (71785 entries) as a database, adopting the following MASCOT search parameters: trypsin, oxidation (M), two missed cleavages allowed for trypsin digestion. Peptide tolerance was set at 10 ppm for MS and at 20 milli-mass units for MS/MS. Scaffold software was used to validate MS/MS based peptide and protein identifications. Peptide and protein identifications were accepted to achieve an FDR less than 1,0% and proteins contained at least 1 identified peptide.

Accessions codes

PP1αT320E was crystallized and the structure solved as described in the according section. The structure presented herein has been deposited at the PDB with the ID 6ZK6.

Supporting References

[1] F. Salvi, M. Trebacz, T. Kokot, B. Hoermann, P. Rios, O. Barabas, M. Köhn, *FEBS Lett.* **2018**, *592*, 4028–4038.

[2] W. Kabsch, *Acta Crystallogr. Sect. D Biol. Crystallogr.* **2010**, *66*, 125–132.

[3] P. Evans, *Acta Crystallogr. Sect. D Biol. Crystallogr.* **2006**, *62*, 72–82.

[4] A. J. McCoy, *Acta Crystallogr. Sect. D Biol. Crystallogr.* **2007**, *63*, 32–41.

[5] M. D. Winn, C. C. Ballard, K. D. Cowtan, E. J. Dodson, P. Emsley, P. R. Evans, R. M. Keegan, E. B. Krissinel, A. G. W. Leslie, A. McCoy, et al., *Acta Crystallogr. Sect. D Biol. Crystallogr.* **2011**, *67*, 235–242.

[6] A. A. Vagin, R. A. Steiner, A. A. Lebedev, L. Potterton, S. McNicholas, F. Long, G. N. Murshudov, *Acta Crystallogr. Sect. D Biol. Crystallogr.* **2004**, *60*, 2184–2195.

[7] P. D. Adams, P. V. Afonine, G. Bunkóczi, V. B. Chen, I. W. Davis, N. Echols, J. J. Headd, L. W. Hung, G. J. Kapral, R. W. Grosse-Kunstleve, et al., *Acta Crystallogr. Sect. D Biol. Crystallogr.* **2010**, DOI 10.1107/S0907444909052925.

[8] P. Emsley, B. Lohkamp, W. G. Scott, K. Cowtan, *Acta Crystallogr. Sect. D Biol. Crystallogr.* **2010**, *66*, 486–501.

[9] M. Beullens, A. Van Eynde, W. Stalmans, M. Bollen, *J. Biol. Chem.* **1992**.

[10] B. Lesage, M. Beullens, L. Pedelini, M. A. Garcia-Gimeno, E. Waelkens, P. Sanz, M. Bollen, *Biochemistry* **2007**, *46*, 8909–8919.

[11] A. Hendrickx, M. Beullens, H. Ceulemans, T. Den Abt, A. Van Eynde, E. Nicolaescu, B. Lesage, M. Bollen, *Chem. Biol.* **2009**, *16*, 365–371.

[12] A. DeGuzman, E. Y. C. Lee, in *Methods Enzymol.*, **1988**, pp. 356–368.
